# Enhancing oxidation resistance of Cu(I) by tailoring microenvironment in zeolites for efficient adsorptive desulfurization

**DOI:** 10.1038/s41467-020-17042-6

**Published:** 2020-06-25

**Authors:** Yu-Xia Li, Jia-Xin Shen, Song-Song Peng, Jun-Kai Zhang, Jie Wu, Xiao-Qin Liu, Lin-Bing Sun

**Affiliations:** State Key Laboratory of Materials-Oriented Chemical Engineering, Jiangsu National Synergetic Innovation Center for Advanced Materials (SICAM), College of Chemical Engineering, Nanjing Tech University, 30 South Puzhu Road, 211816 Nanjing, China

**Keywords:** Green chemistry, Inorganic chemistry

## Abstract

The zeolite Cu(I)Y is promising for adsorptive removal of thiophenic sulfur compounds from transportation fuels. However, its application is seriously hindered by the instability of Cu(I), which is easily oxidized to Cu(II) even under atmospheric environment due to the coexistence of moisture and oxygen. Here, we report the adjustment of zeolite microenvironment from hydrophilic to superhydrophobic status by coating polydimethylsiloxane (yielding Cu(I)Y@P), which isolates moisture entering the pores and subsequently stabilizes Cu(I) despite the presence of oxygen. Cu(I) in Cu(I)Y@P is stable upon exposure to humid atmosphere for 6 months, while almost all Cu(I) is oxidized to Cu(II) in Cu(I)Y for only 2 weeks. The optimized Cu(I)Y@P material after moisture exposure can remove 532 μmol g^−1^ of thiophene and is much superior to Cu(I)Y (116 μmol g^−1^), regardless of similar uptakes for unexposed adsorbents. Remarkably, Cu(I)Y@P shows excellent adsorption capacity of desulfurization for water-containing model fuel.

## Abstract (web summary)

Zeolite Cu(I)Y is attractive for adsorptive removal of sulfur compounds from fuel, however practical application is limited by instability of Cu(I). Here the authors use a coating to achieve superhydrophobicity in the zeolite, leading to improved Cu(I) stability against oxidation and thiophene removal.

## Introduction

Emission of acidic pollutants like SO_2_ that originate from the combustion of organosulfur compounds in fuels is a serious environmental issue^1–3^. Therefore, removal of organosulfur compounds from transportation fuels has attracted worldwide attention^4–6^. Recently, deep desulfurization becomes more challenging due to even tighter regulations on sulfur contents in commercial fuels^7–9^. Although hydrodesulfurization (HDS) can eliminate thiols and sulfides efficiently, it is less effective for the removal of thiophenic sulfur compounds such as thiophene, benzothiophene (BT), and their derivatives^10–12^. In addition, HDS is generally operated at high temperatures (300 − 350 °C) and high hydrogen pressures (2 − 10 MPa), and even at harsher conditions, to meet the regulations with lower sulfur contents^13–15^. Among alternatives for deep desulfurization, adsorption desulfurization (ADS) receives much attention because it can remove thiophenic sulfur compounds selectively under mild conditions. It is known that the ADS performance is highly dependent on the type of adsorbents^16–18^, and substantial progresses have been achieved on the preparation of adsorbents for ADS^9,19,20^.

Various adsorbents including activated carbons^21^, zeolites^22,23^, and metal-organic frameworks (MOFs)^24–26^ have been developed for ADS. Cu(I)-containing adsorbents^27–30^ are of great interests owing to the π-complexation interaction between Cu(I) and thiophenic sulfur compounds. It has been demonstrated that Cu(I)-exchanged Y zeolite, namely Cu(I)Y, exhibits unique faujasite (FAU) pore structure, stable inorganic frameworks, and abundant Cu(I) sites^31^. These properties endow Cu(I)Y with good ADS performance with regard to uptake and selectivity, making it highly promising for deep desulfurization of transportation fuels^32^. Nevertheless, the practical application of Cu(I)Y is seriously hindered by the instability of Cu(I) that is easily oxidized to Cu(II) even under atmospheric environment due to the coexistence of moisture and oxygen^33,34^. Cu(I) can capture thiophenic compounds through π-complexation, a special interaction between Cu(I) and the π-orbital of thiophenics; however, Cu(II) does not show a noticeable adsorption capacity because Cu(II) does not give such a π-complexation interaction^27^. During the complexation, Cu(I) can form the usual *σ* bonds based on their *s*-orbitals and, in addition, their *d*-orbitals can back-feed electrons to the antibonding π-orbitals of the thiophenic compounds. The preparation, storage, and utilization of Cu(I) have to be conducted in the absence of air, which leads to the difficulty in operation and significant increase of costs. It is reported that the oxidation of Cu(I) to Cu(II) by oxygen does not take place at room temperature in a dry environment, but easily occurs when oxygen is adsorbed on hydrated surfaces^33^. Therefore, in order to stabilize Cu(I), it is necessary to isolate Cu(I) sites contacting with either moisture or oxygen. In contrast to avoiding contact with oxygen, the preclusion of moisture seems earlier to realize. In addition, water is inevitable in commercial fuels. For instance, the water content of BP commercial diesels is in the range of 100 and 500 ppmw (parts per million by weight)^35^. Such water in fuels not only accelerates the oxidation of Cu(I), but also competes with thiophenic sulfur compounds to interact with active sites in adsorbents^36^. Hence, from the viewpoint of practical application, it is extremely desirable to develop an approach to tune the nature of Cu(I)Y zeolite, so that the accessibility of Cu(I) sites to moisture can be excluded and the stability of Cu(I) is thus improved.

**Fig. 1:**
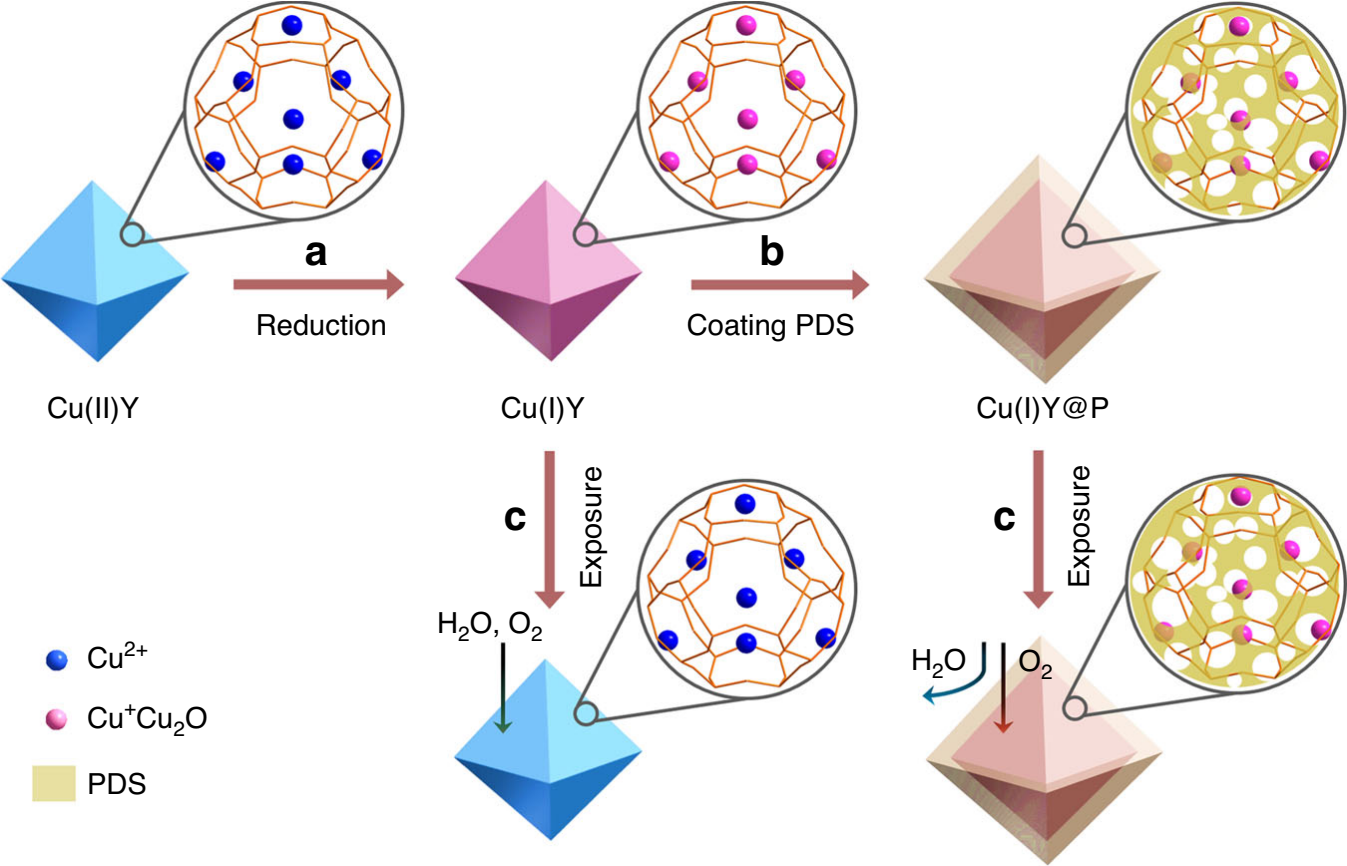
Construct superhydrophobic microenvironment and enhance Cu(I) oxidation resistance. **a** Selective reduction of Cu(II)Y to Cu(I)Y; **b** Creation of superhydrophobic surface via coating PDS on Cu(I)Y; **c** Enhancement of oxidation resistance of Cu(I) after PDS coating upon exposure to humid atmosphere. The access of moisture to Cu(I) sites is excluded by superhydrophobic surface, and oxidation is hindered despite the presence of oxygen.

Here we report a strategy of tailoring the Cu(I)Y microenvironment from hydrophilic to superhydrophobic by coating polydimethylsiloxane (PDS), producing the materials denoted as Cu(I)Y@P (Fig. 1). This isolates moisture entering the pores and subsequently stabilizes the Cu(I) despite the presence of oxygen. The results show that Cu(I) in Cu(I)Y@P is stable upon exposure to humid atmosphere with 75% relative humidity (RH) for 4320 h (6 months), while almost all Cu(I) is oxidized to Cu(II) in uncoated Cu(I)Y for only 336 h (2 weeks). The optimized Cu(I)Y@P material after moisture exposure can remove 532 μmol g^−1^ of thiophene, which is obviously higher than that of Cu(I)Y (116 μmol g^−1^), regardless of similar uptakes for unexposed adsorbents. It is worth noting that Cu(I)Y@P shows excellent ADS capacity for water-containing model fuel and is superior to all adsorbents reported so far. Furthermore, the adsorbent Cu(I)Y@P can be recycled without any loss in activity, whereas only 3% of adsorption capacity is retained after four cycles for uncoated Cu(I)Y. The good oxidation resistance, adsorption capacity, and recyclability make our adsorbents highly promising in practical ADS application.

## Results

### Effect of PDS coating on structural and surface properties

Cu(II)Y was prepared by ion exchange of the zeolite NaY with copper(II) nitrate and reduced to Cu(I)Y selectively via vapor-induced reduction (VIR) in term of our previous reports^37,38^. To tailor the microenvironment of Cu(I)Y, PDS was chosen as a typical compound for the surface hydrophobic modification through chemical vapor deposition (CVD, Fig. 1). The amount of PDS coated is tunable by varying the CVD time from 15 min to 30 min and 45 min, producing the samples denoted as Cu(I)@P(*n*), where *n* ranged from 2.3% to 3.1% and 4.0% corresponds to the weight percentage of PDS calculated by elemental analysis (Supplementary Table 1).

**Fig. 2:**
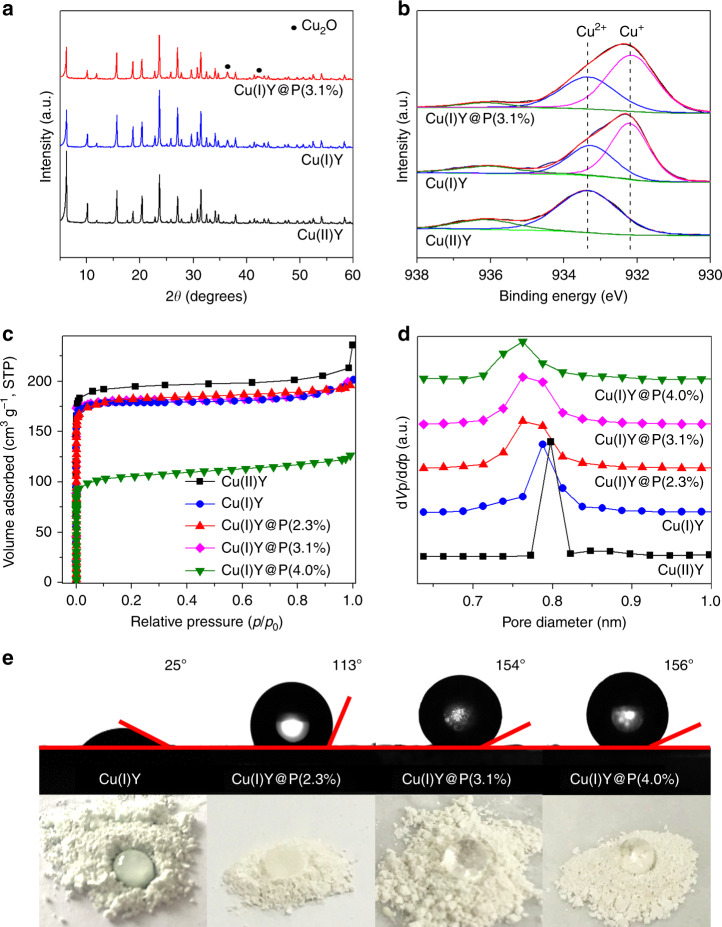
Characterization of zeolites before and after coating PDS. **a** XRD patterns and **b** XPS spectra of Cu(II)Y, Cu(I)Y, and Cu(I)Y@P(3.1%); **c** N_2_ adsorption isotherms and **d** pore size distributions of Cu(II)Y, Cu(I)Y, and Cu(I)Y@PDS; **e** Static water contact angles and pictures of different samples with a drop of water.

The reduction of Cu(II) to Cu(I) in the zeolite was first examined. For Cu(II)Y before reduction, its X-ray diffraction (XRD, Fig. 2a) pattern is identical to that of pristine NaY (Supplementary Fig. 1), indicating that the crystalline structure of zeolite Y is well preserved during ion exchange. After reduction, two new diffraction peaks at 36.4° and 42.3° ascribed to Cu_2_O appear in Cu(I)Y^39^. In the case of Cu(I)Y@P(3.1%), the characteristic peaks of Cu_2_O can also be observed. This suggests that Cu(II) ions are reduced to Cu_2_O in addition to Cu(I) ions^39^. The successful reduction of Cu(II) to Cu(I) is also confirmed by UV–vis spectra (Supplementary Fig. 2). The observed absorption peak ranging from 700 to 800 nm in Cu(II)Y corresponds to the *d*–*d* transition of Cu(II)^40^. After VIR, the intensity of band around 800 nm declines slightly. This is caused by the incomplete reduction of Cu(II) and oxygen-to-metal charge transfer of Cu(I) in the 6–6 secondary building unit of zeolite^41^. Meanwhile, two new absorption peaks at 350 and 450 nm readily attributed to the charge transfer transitions (3d^10^ → 3d^9^4s^1^) of Cu(I) in the zeolite matrix can be clearly identified, which is absent in Cu(II)Y^42,43^. Inductively coupled plasma results show that the ion-exchange ratio in Cu(II)Y is 79%, corresponding to a total Cu(II) content of 1.18 mmol g^−1^. Quantitative analysis by X-ray photoelectron spectroscopy (XPS) shows that after reduction the Cu(I) content in Cu(I)Y@P(3.1%) is 61.5%, which is analogous to that in Cu(I)Y (60.8%, Fig. 2b). In situ IR analysis using CO as a probe was employed to quantify the amount of single Cu^+^ species and the results are shown in Supplementary Fig. 3. No peak is observed in in situ IR spectrum of Cu(II)Y, indicating that Cu^2+^ site does not work as an active site. Single Cu^+^ species located at different exchange sites in zeolites are characterized by two overlapping bands at 2145 and 2160 cm^−1^, while a shoulder at 2110 cm^−1^ is assigned to Cu_2_O species^44^. The results obtained from curve-fitting present that the content of isolated Cu^+^ is 69% of the total amount of Cu(I) species (0.49 mmol g^−1^), which works as the charge compensation ions for neutralizing negative charges emanating from the zeolite-lattice. In addition to Cu^+^, protons (H^+^) generated during reduction work as the charge compensation ions in the sample as demonstrated in previous studies^39,45,46^. Temperature-programmed desorption profile of thiophene on Cu(I)Y is shown in Supplementary Fig. 4. Two thiophene desorption peaks centered at 72 and 127 °C can be observed. Both are caused by the adsorbate–adsorbent interaction. Although π-complexation interaction as a subclass of chemical interaction is stronger than physisorption, the π-complexation bonds are weak enough as to be broken by increasing temperature^47^. The desorption at the higher temperature corresponds to the π-complexation interaction. These results suggest that Cu(II) is converted to Cu(I) successfully by use of the VIR and the formed Cu(I) is well maintained after coating PDS.

The structure of zeolite after PDS coating was then investigated. Quite similar XRD patterns (Fig. 2a) and field-emission scanning electron microscope (Supplementary Fig. 5) images can be observed for Cu(I)Y and Cu(I)Y@P, suggesting that PDS coating does not affect the crystalline structure and morphology of zeolite. All of the bands in Fourier transform infrared spectra of the parent zeolite are maintained after PDS coating (Supplementary Fig. 6). In the meanwhile, the band at 1264 cm^‒1^ originated from CH_3_–Si vibration is visible in Cu(I)Y@P^48,49^, and becomes stronger with the increase of PDS amount. The PDS layer can be directly observed in high-resolution transmission electron microscopy images (Supplementary Fig. 7). The layer locates on the external surface of zeolite and the thickness ranges from 1.3 to 3.8 nm. N_2_ adsorption isotherms and pore size distributions show that the pore structure of zeolite is well retained after coating <3.1% PDS (Fig. 2c, d). Further increasing the coating amount to 4.0%, the N_2_ uptake decreases sharply. Further calculation shows that the Brunauer–Emmett–Teller surface area of Cu(I)Y@P(2.3%) and Cu(I)Y@P(3.1%) is 744 and 728 m^2^ g^−1^, which is comparable with that of uncoated Cu(I)Y (745 m^2^ g^−1^, Supplementary Table 1), indicating the good permeability of the thin PDS layer and/or incomplete surface coating. However, the surface area of Cu(I)Y@P(4.0%) is only 410 m^2^ g^−1^; this is because excessive PDS on the surface interrupts the accessibility of internal pores of Cu(I)Y. Based on the above results, it is clear that the crystalline structure and morphology of Cu(I)Y zeolite is well preserved after PDS coating and that the coating of suitable amount of PDS does not affect the accessibility of inner pores.

The surface wettability of Cu(I)Y and Cu(I)Y@PDS was evaluated by water contact-angle measurements (Fig. 2e). Cu(I)Y gives a water contact angle of 25°, indicative of the hydrophilic nature of the pristine material. After coating 2.3% PDS on Cu(I)Y, the water contact angle increases to 113°, reflecting that the surface transformed from hydrophilic to hydrophobic. When the coating amount of PDS is larger than 3.1%, the contact angle is higher than 154°, indicating the superhydrophobic feature of Cu(I)Y@P(3.1%) and Cu(I)Y@P(4.0%). Moreover, the water droplets on the surface of these two samples are spherical in shape and can roll easily. This demonstrates that, through coating a small amount of PDS, it is sufficient to achieve super-hydrophobicity on Cu(I)Y. Water adsorption isotherms of Cu(I)Y before and after PDS coating are presented in Supplementary Fig. 8. After PDS coating, the adsorption capacity on water decreases obviously, which confirms the hydrophobic characteristic of Cu(I)Y@P^25,50^. By combining the results of pore structure and wettability, it is obvious that Cu(I)Y@P(3.1%) is an optimal sample which will be used for the detailed evaluation of stability and adsorption performance.

Based on the aforementioned results, it is conclusive that PDS is successfully coated on the external surface of zeolite Cu(I)Y, and the thickness of PDS layer is tunable by changing the deposition time. After coating PDS, the Cu(I) sites and crystalline structure of the parent Cu(I)Y are well maintained. The coating of suitable amount of PDS leads to the transformation of surface nature from hydrophilic to superhydrophobic, while the internal pores of zeolite keeps highly accessible.

### Oxidation resistance of Cu(I) in superhydrophobic space

**Fig. 3:**
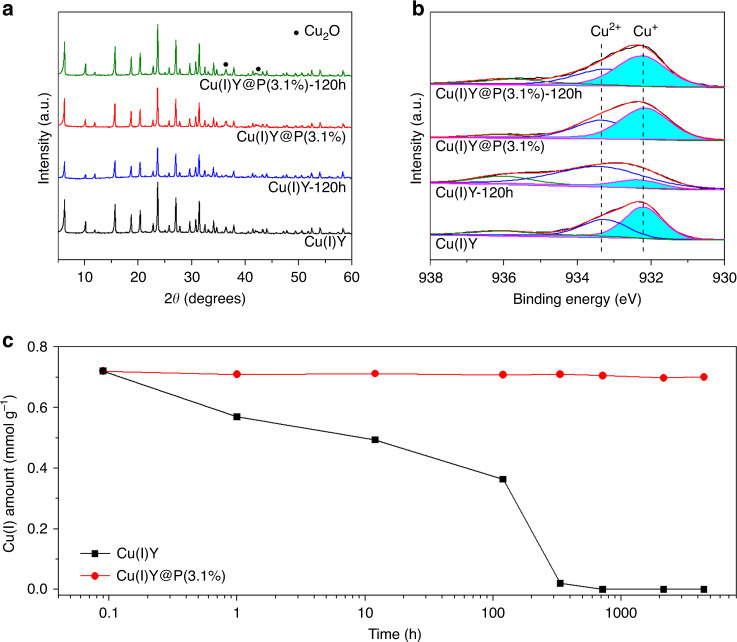
Oxidation resistance of Cu(I). **a** XRD patterns and **b** XPS spectra of Cu(I)Y and Cu(I)Y@P(3.1%) after exposure to humid atmosphere (RH = 75%) for 120 h; **c** Cu(I) content of Cu(I)Y and Cu(I)Y@P(3.1%) after exposure to humid atmosphere (RH = 75%) for 6 months.

By tailoring the zeolite microenvironment from hydrophilic to superhydrophobic, the access of moisture to the pores is hindered. Because the oxidization of Cu(I) to Cu(II) only occurs in the coexistence of moisture and oxygen, the interception of moisture should enhance the oxidation resistance of Cu(I) despite the presence of oxygen. To examine the oxidation resistance, Cu(I)-containing samples were exposed to the humid atmosphere with 75% RH to accelerate the oxidation of Cu(I). It has been exposed for 4320 h (6 months) when the paper is ready for submission, and Cu(I) contents of the samples upon exposure for different time were monitored. Typical results after exposure for 120 h are displayed in Fig. 3 and the obtained samples were denoted as Cu(I)Y-120 h and Cu(I)Y@P(3.1%)-120 h, respectively. In the XRD pattern of Cu(I)Y-120 h, the characteristic peaks of Cu_2_O at 36.4° and 42.3° disappear, indicating the oxidation of Cu(I). On the contrary, the diffraction peaks of Cu_2_O in the XRD pattern of Cu(I)Y@P(3.1%)-120 h are comparable with unexposed Cu(I)Y@P(3.1%). XPS analysis shows that about 50% of Cu(I) is oxidized to Cu(II) in Cu(I)Y-120 h, while the content of Cu(I) in Cu(I)Y@P(3.1%) keeps constant after exposure to humid atmosphere (Fig. 3b). These results evidence that PDS coating is efficient in preventing Cu(I) from oxidizing.

Resistance of Cu(I) oxidation in long time in humid atmosphere is displayed in Fig. 3c. Regardless of the same initial Cu(I) content in Cu(I)Y and Cu(I)Y@P(3.1%), rather different alteration behavior is detected upon exposure in humid atmosphere. After exposure for 12 h, the content of Cu(I) in Cu(I)Y decreases obviously from 0.72 to 0.49 mmol g^−1^. With prolonging exposure time, the decline of Cu(I) content is more evident. After 336 h (2 weeks), the Cu(I) content in Cu(I)Y is only 0.02 mmol g^−1^, suggesting that almost all Cu(I) is oxidized to Cu(II). On the contrary, Cu(I) content of Cu(I)Y@P(3.1%) does not alter at all after exposure in humid atmosphere even for 4320 h (6 months). Apparently, Cu(I)Y@P(3.1%) exhibits excellent long-term stability against Cu(I) oxidation, which is caused by the superhydrophobic microenvironment constructed by PDS coating. The entrance of moisture to internal pores is forbidden and the oxidation of Cu(I) is terminated at the first step despite the presence of oxygen. The oxidation resistance of Cu(I)Y@P endows them with excellent ADS performance as shown below.

### Adsorptive desulfurization performance

**Fig. 4:**
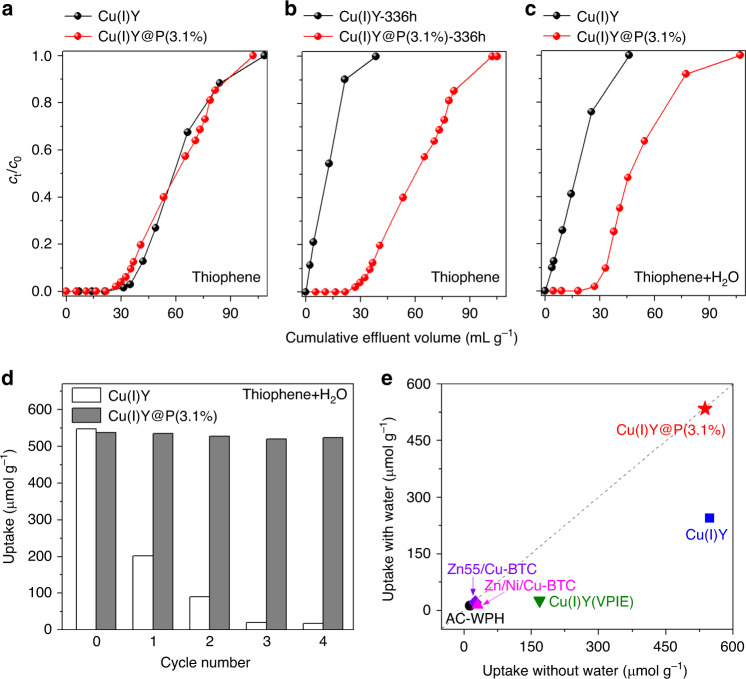
Adsorptive desulfurization performance. Breakthrough curves of the model fuel containing 550 ppmw thiophene over Cu(I)Y and Cu(I)Y@P(3.1%) **a** before and **b** after exposure to humid atmosphere (RH = 75%) for 336 h; **c** Breakthrough curves of the model fuel containing 550 ppmw thiophene with the addition of 300 ppmw H_2_O over Cu(I)Y and Cu(I)Y@P(3.1%); **d** Recyclability of Cu(I)Y and Cu(I)Y@P(3.1%) in the model fuel containing 550 ppmw thiophene with addition of 300 ppmw H_2_O; **e** Comparison of desulfurization capacity of different adsorbents in the fuels with and without addition of H_2_O.

ADS performance of the obtained materials was first assessed under normal operating conditions and moisture/water was not involved. A typical aromatic sulfur compound, thiophene, was initially used for evaluation. Cu(II)Y is capable of capturing 62 μmol g^−1^ of thiophene (Supplementary Fig. 9). It is noticeable that, after reduction, the resultant Cu(I)Y displays obviously enhanced adsorption capacity of 548 μmol g^−1^, due to the π-complexation interaction between Cu(I) and thiophene. Through the π-complexation mechanism, the empty *s*-orbital of Cu(I) species forms a *σ* bond with π-electrons of thiophene and, in addition, *d*-orbital of Cu(I) species forms a π back-donation with antibonding π-orbitals (π*) of thiophene. IR spectra of Cu(I)Y and Cu(II)Y after adsorption with thiophene are shown in Supplementary Fig. 10. The adsorption of thiophene on zeolite triggers vibrational stretches and the peaks at 1479 and 1400 cm^−1^ are attributed to the symmetrical C=C stretching vibration of thiophene on Cu(II)Y^23,51^. For thiophene adsorbed on Cu(I)Y, these two peaks have been shifted to a lower wavenumber suggesting thiophene ring stacks above an active site via π-complexation^23^. In addition to the bands derived from adsorbed thiophene, new bands at 1325 and 1352 cm^−1^ originated from the π-complexation interaction between thiophene and Cu(I) via the sulfur-metal mode are observed^52^. Therefore, π-complexes are formed between Cu(I) species and thiophene, which leads to selective adsorption of thiophenic sulfur compounds. Of course, in addition to π-complexation interaction, acid sites and pore filling also contribute to adsorptive desulfurization performance. Considering that there is 0.72 mmol g^−1^ of Cu(I) formed in zeolite after reduction, in which isolated Cu^+^ is 0.49 mmol g^−1^ and the other is Cu_2_O, and this corresponds to a ratio of thiophene/Cu(I) of 0.76. The adsorbed thiophene is 548 μmol g^−1^, which is higher than the amount of Cu^+^ but lower than that of total Cu(I) amount (Cu^+^ and Cu_2_O). This indicates that Cu^+^ and some Cu_2_O act as active sites whereas other Cu_2_O aggregated in pores are inaccessible to adsorbates. The PDS-coated materials Cu(I)Y@P(2.3%) and Cu(I)Y@P(3.1%) can capture 529 and 537 μmol g^−1^ of thiophene, respectively, which is comparable with uncoated Cu(I)Y (Fig. 4a and Supplementary Fig. 11). The increase of coating amount to 4.0% leads to the decrease in ADS capacity and the adsorption amount of thiophene on Cu(I)Y@P(4.0%) is 424 μmol g^−1^. This is because the pores of zeolite are partially blocked by the excessive PDS as demonstrated by N_2_ adsorption data shown above, resulting in diffusion resistance and inaccessibility of some Cu(I) sites. Apart from thiophene, two typical aromatic sulfur compounds BT and 4,6-dimethyldibenzothiphene (DMDBT) with larger molecular sizes were also tested (Supplementary Fig. 12). The molecular size of thiophene, BT, and DMDBT is 0.56 nm × 0.77 nm, 0.65 nm ×  0.89 nm, and 0.78 nm × 1.23 nm, respectively. The size of zeolite micropores is centered at 0.8 nm, and thus these aromatic sulfur compounds can enter the pores. The results show that Cu(I)Y@P(3.1%) is efficient in capturing BT (1053 μmol g^−1^) and DMDBT (1123 μmol g^−1^) as well. Such capacity is higher than that for thiophene, which is due to stronger π-complexation interaction between Cu(I) and sulfur compounds with more aromatic rings^53^. The presence of additional aromatic rings in BT and DMDBT can increase the π-electron number, enhancing the interaction of thiophenic molecules with Cu(I) sites^54^. Aromatics exist in fuels and can compete with thiophenic sulfur compounds to be adsorbed on active sites. To study the selective adsorption capacity of adsorbents, *tert*-butyl benzene as a competitive adsorbate was added to model fuel. Cu(I)Y and Cu(I)Y@P(3.1%) show similar adsorption capacity on thiophene, and the adsorption capacity of both adsorbents decrease in the presence of *tert*-butyl benzene (Supplementary Fig. 13). This is caused by competitive adsorption between *tert*-butyl benzene and thiophene. In comparison with Cu(I)Y, Cu(I)Y@P(3.1%) can adsorb more thiophene in the presence of *tert*-butyl benzene. Therefore, Cu(I)Y@P(3.1%) is more selective on the adsorption of thiophene in the presence of *tert*-butyl benzene.

The adsorbate–adsorbent interaction is of prime importance and closely related to adsorption capacity; without adsorbate–adsorbent interaction, the adsorption capacity of an adsorbent would be very scarce, if any^55^. As a kind of adsorbate–adsorbent interaction, π-complexation interaction is generally stronger than physisorption, yet weak enough to be reversible^47^. In addition to π-complexation interaction, physisorption such as pore filling also contributes to adsorptive desulfurization performance^56^. The total adsorption capacity may be caused by the combination of different kinds of adsorbate–adsorbent interactions, while π-complexation interaction plays a predominant role in the present study. Based on the aforementioned results, a suitable amount of PDS does not affect the accessibility of inner pores and Cu(I) active sites. Moreover, the obtained Cu(I)Y@P materials possess the same ratio of ion exchange and Cu(I) as uncoated Cu(I)Y. Therefore, Cu(I)Y@P with a suitable amount of PDS possess comparable adsorbate–adsorbent interaction with Cu(I)Y. For BT and DMDBT, the steric hindrance has an effect on the adsorption amount, while the adsorbate–adsorbent interaction is another factor influencing the adsorption amount. The effect of steric hindrance may become predominant for physisorption, while adsorbate–adsorbent interaction plays a main role in chemisorption^47^. The presence of additional aromatic rings in BT and DMDBT increases the π-electron number, thus enhancing the interaction of thiophenic molecules with Cu(I) sites^54^. Moreover, the adsorption energy is employed to further evaluate the interaction with aromatic sulfur compounds^57,58^. As shown in Supplementary Table 2, the adsorption energies of thiophene, BT, and DMDBT vary from 21.4 to 23.6 kcal mol^−1^, which is a moderate strength interaction typical for π-complexation interaction^59^. It also can be seen that the adsorption energy for thiophenic sulfur compounds decreases in the order of DMDBT > BT > thiophene. In short, benzene rings have an enhancement effect on the π-complexation interaction of different thiophenic sulfur compounds with Cu(I)Y. This can interpret the adsorption capacity of Cu(I)Y on three thiophenic sulfur compounds.

ADS performance of the materials after exposure to humid atmosphere was then evaluated. The thiophene uptakes on Cu(I)Y and Cu(I)Y@P(3.1%) after exposure for different time were monitored. For Cu(I)Y after 120 h exposure, Cu(I)Y-120h can only capture 176 μmol g^−1^ of thiophene (Supplementary Fig. 14), which is much lower than unexposed Cu(I)Y (548 μmol g^−1^). Further increasing the exposure time to 336 h, the adsorption capacity on the obtained material Cu(I)Y-336 h decreases to 116 μmol g^−1^ (Fig. 4b). This is due to the oxidation of Cu(I) to Cu(II) in the zeolite Cu(I)Y upon moisture exposure as demonstrated above. The exposed material containing Cu(II) lacks π-complexation interaction with thiophene, thus leading to sharply decreased adsorption capacity. In the case of PDS-coated material Cu(I)Y@P(3.1%), the effect of exposure to humid atmosphere on ADS performance is totally different from uncoated Cu(I)Y. After exposure for 120 h, the thiophene uptake is 532 μmol g^−1^, which is comparable the unexposed Cu(I)Y@P(3.1%) (537 μmol g^−1^). Further increasing the exposure time to 336 h, the adsorption capacity of Cu(I)Y@P(3.1%) does not decline at all. These results prove that the superhydrophobic microenvironment effectively prevents instable Cu(I) sites from oxidizing and retains the high activity of PDS-coated Cu(I)Y upon exposure to humid atmosphere.

Based on the aforementioned results, the model fuel with the addition of 300 ppmw water was directly utilized to investigate the ADS performance, referring to the water content in commercial fuels (Fig. 4c). Cu(I)Y can capture 244 μmol g^−1^ of thiophene from the hydrated fuel, whereas the thiophene uptake is 548 μmol g^−1^ from the anhydrous fuel, indicating 55% loss of activity in the presence of water. In sharp contrast, water has a negligible effect on the adsorption capacity of PDS-coated material Cu(I)Y@P(3.1%). The thiophene uptake on Cu(I)Y@P(3.1%) from the hydrated fuel is 535 μmol g^−1^, which is analogous to that from the anhydrous fuel (537 μmol g^−1^). The recyclability of adsorbents in the desulfurization from the hydrated fuel was examined due to the importance in practical applications (Fig. 4d). It is noticeable that no obvious loss in activity is observed for Cu(I)Y@P(3.1%) during cycling, while Cu(I)Y can only remove 18 μmol g^−1^ of thiophene and loses 97% of activity after four cycles. These results demonstrate excellent recyclability and oxidation resistance of PDS-coated Cu(I)Y, whereas the presence of a trace of water compromises the activity of uncoated Cu(I)Y dramatically. Two factors are responsible for the loss of activity for Cu(I)Y. On one hand, water can compete with thiophene to interact with active sites in zeolite; on the other hand, water initiates the oxidization of Cu(I), forming Cu(II) which is inactive for thiophene capture.

The ADS performance of resultant adsorbents for hydrated fuels is compared with those reported in literature (Fig. 4e and Supplementary Table 3). For activated carbon and polymetallic MOFs, the adsorption capacity on anhydrous fuels is low and <30 μmol g^−1^, and the uptakes decrease by 10–50% in the existence of a trace amount of water^60–62^. For Cu(I)Y(VPIE) prepared by the vapor-phase ion-exchange method, the adsorption capacity is 168 μmol g^−1^; nonetheless, such capacity drops to 26.5 μmol g^−1^ with the addition of 300  ppmw to fuel^35^. Noteworthily, our adsorbent Cu(I)Y@P(3.1%) has an uptake of 537 μmol g^−1^ for anhydrous fuel, and such an uptake does not decrease at all in the presence of water. According to the comparison, it is apparent that the PDS-coated Cu(I)Y exhibits excellent ADS performance especially for hydrated fuels. By coating PDS, the competitive adsorption of water with thiophenic sulfur compounds and the oxidization of active sites in the presence of water can be avoided, making the present adsorbents highly promising for practical ADS applications.

## Discussion

We have demonstrated the successful coating of PDS on Cu(I)Y, and with tuning the coating amount, the active Cu(I) sites, crystalline structure, and the porosity of zeolite can be well maintained while the hydrophilic surface was transformed to superhydrophobic one. The superhydrophobic surface obstructs the access of moisture into pores, thus avoiding the oxidation of Cu(I) despite the presence of oxygen, with considering that the oxidization of Cu(I) to Cu(II) only took place in the coexistence of moisture and oxygen. The experimental results show that the Cu(I) content in PDS-coated Cu(I)Y can remain stable in humid atmosphere for 6 months, which is different from uncoated Cu(I)Y whose Cu(I) is almost oxidized to Cu(II) within 2 weeks. Note that Cu(I)Y@P gives excellent ADS capacity for hydrated fuel and is superior to all adsorbents reported so far. Moreover, the Cu(I)Y@P exhibits good reusability for hydrated fuel and no loss in activity is observed during recycling, while only 3% of adsorption capacity is maintained after four cycles for uncoated Cu(I)Y. The antioxidation of Cu(I)Y@P makes the storage and utilization of Cu(I)-containing adsorbents much easier. The resistance to oxidation, along with good adsorption capacity and recyclability for hydrated fuels, makes the present adsorbents highly promising in practical ADS application. Such a facile strategy might open up a new avenue for the fabrication of stable functional materials for various applications.

## Methods

### Materials preparation

Cu(II)Y zeolite was prepared by ion exchange of NaY with aqueous Cu(NO_3_)_2_ solution and reduced to Cu(I)Y by the VIR method^37,38^ (see Supplementary Information for details). The coating of PDS on Cu(I)Y was carried out by the CVD technique, producing Cu(I)Y@P(*n*), where *n* corresponds to the weight percentage of PDS.

### ADS experiments

The model fuel used for experiments was prepared by mixing thiophene, BT, or DMDBT with isooctane, and the sulfur content was ~550 ppmw. In order to investigate the competitive adsorption between aromatics and thiophenic sulfur compounds, 10 wt% of *tert*-butyl benzene was mixed with model fuel containing 550 ppmw thiophene in isooctane. Moisture is present in all commercial fuels. In order to test the effect of moisture on desulfurization capacity, the model fuel containing 550 ppmw thiophene in isooctane was mixed and thoroughly agitated with 300 ppmw water. Adsorptive experiments were carried out in the dynamic conditions and performed in a fixed bed set up at room temperature. The model fuel was allowed to contact the adsorbent pumped up with a mini creep pump at the rate of 3 mL h^−1^. Adsorbents were pretreated in flowing Ar at 150 °C for 6 h followed by cooling to room temperature. Effluent solutions were collected periodically until saturation was reached. The sulfur content of effluent solutions was analyzed using a Varian 3800 gas chromatograph (GC) equipped with a pulsed-flame photometric detector. A calibration curve was prepared to verify the GC results. Breakthrough curves were generated by plotting the normalized sulfur concentration versus the cumulative fuel volume, which was normalized by the adsorbent weight. The normalized sulfur concentration (*c*_t_/*c*_0_) was obtained by measuring the ratio of the detected sulfur content (*c*_t_) to that of the initial sulfur content (*c*_0_). The adsorption capacity *q* (mmol g^−1^) was obtained from integral calculus as shown in Eq. (1).

1 $$q\,=\,\frac{v}{m}\frac{{\rho X_0}}{M}\mathop {\int}\limits_0^t {\left( {1\,-\,\frac{{c_{\rm{t}}}}{{c_0}}} \right)} d_{\rm{t}},$$

where *v* is the feed flow rate (mL min^−1^), *ρ* is the model fuel density (g mL^−1^) at room temperature, *X*_0_ is the total sulfur fraction in the feed, *m* is the weight of the adsorbent (g), and *M* is the molecular weight of sulfur (g mol^−1^). The integral on the right-hand side of Eq. (1) is the area above the breakthrough curves at saturation time *t*; at that time the effluent sulfur concentration was equal to the sulfur concentration in the feed.

## Supplementary information

Supplementary Information

## Data Availability

The data that support the findings of this work are available within the article and its Supplementary Information files. All other relevant data supporting the findings of this study are available from the corresponding author on reasonable request.
