# Supplementary material for: Enhancing oxidation resistance of Cu(I) by tailoring microenvironment in zeolites for efficient adsorptive desulfurization
Source: Nat Commun. 2020 Jun 25;11:3206. doi: 10.1038/s41467-020-17042-6 (PMC7316705; doi:10.1038/s41467-020-17042-6)
Supplement: Supplementary file 1 — Supplementary Information [file 41467_2020_17042_MOESM1_ESM.pdf]

Supplementary Information

Enhancing oxidation resistance of Cu(I) by tailoring microenvironment in zeolites for efficient adsorptive desulfurization

Li et al.

State Key Laboratory of Materials-Oriented Chemical Engineering, Jiangsu National Synergetic Innovation Center for Advanced Materials (SICAM), College of Chemical Engineering, Nanjing Tech University, Nanjing 211816, China.

★Corresponding author: lbsun@njtech.edu.cn

Supplementary Figures

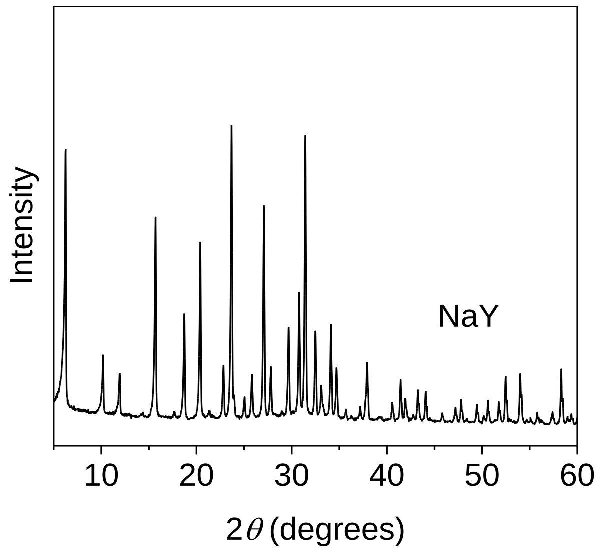

Supplementary Figure 1. XRD pattern of NaY.

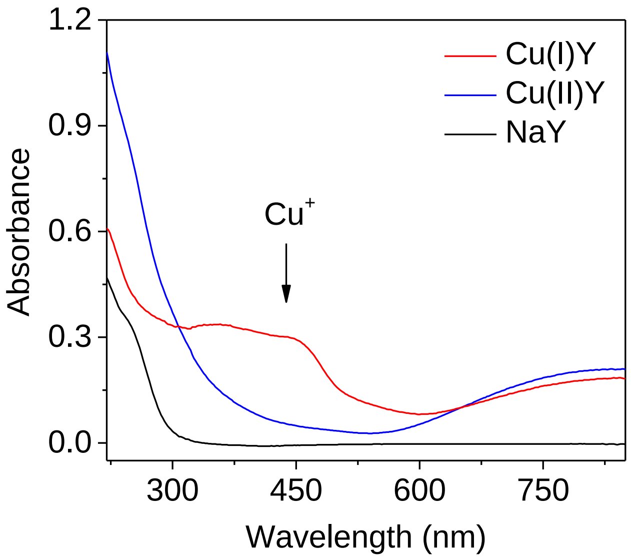

Supplementary Figure 2. UV-vis absorption spectra of NaY, Cu(II)Y, and Cu(I)Y.

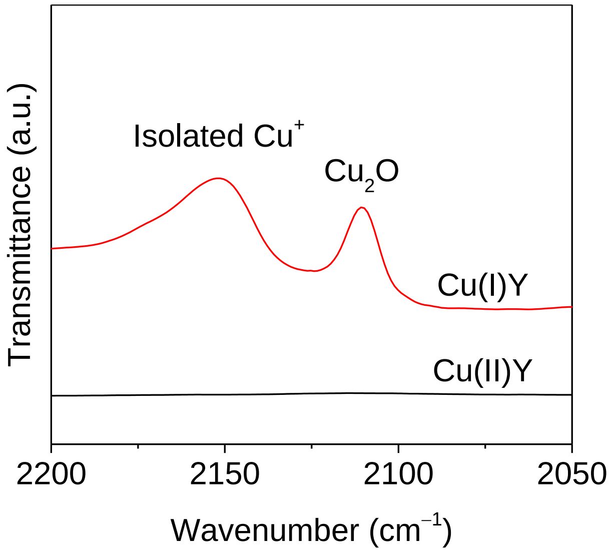

Supplementary Figure 3. DRIFT spectra of CO adsorbed on Cu(II)Y and Cu(I)Y.

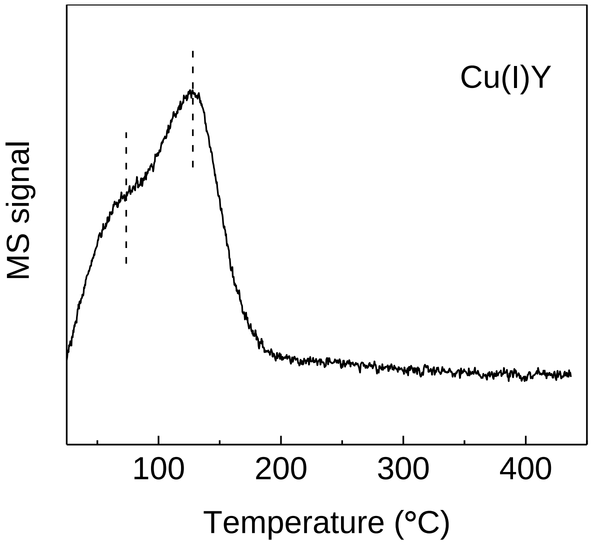

Supplementary Figure 4. TPD profile of thiophene on Cu(I)Y.

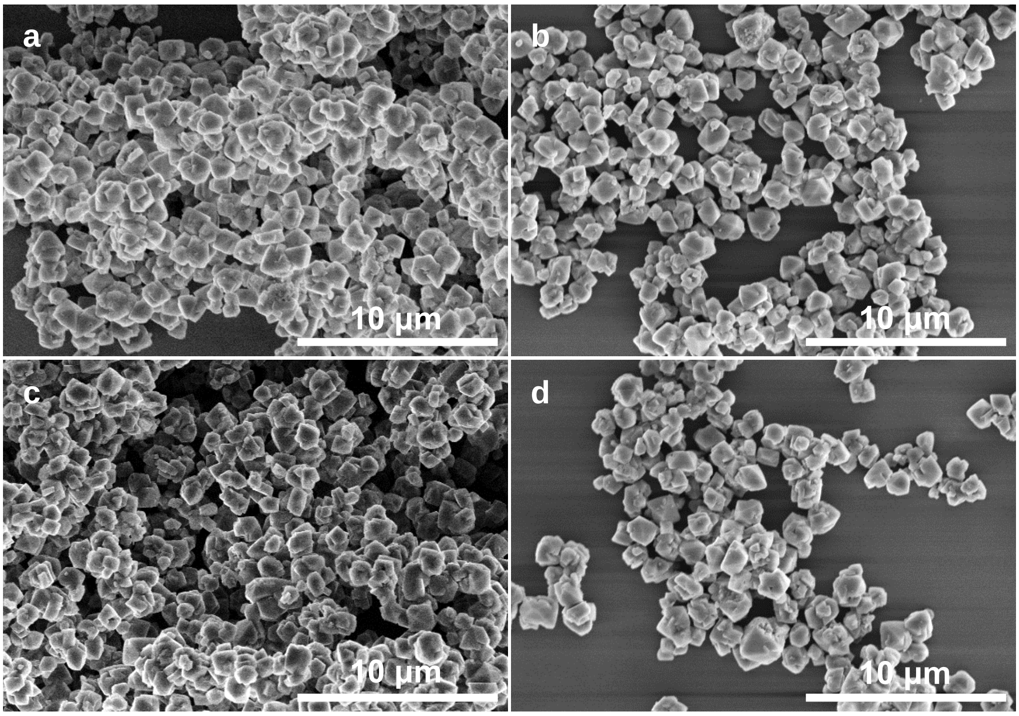

Supplementary Figure 5. SEM images of (a) Cu(I)Y, (b) Cu(I)Y@P(2.3%), (c) Cu(I)Y@P(3.1%), and (d) Cu(I)Y@P(4.0%).

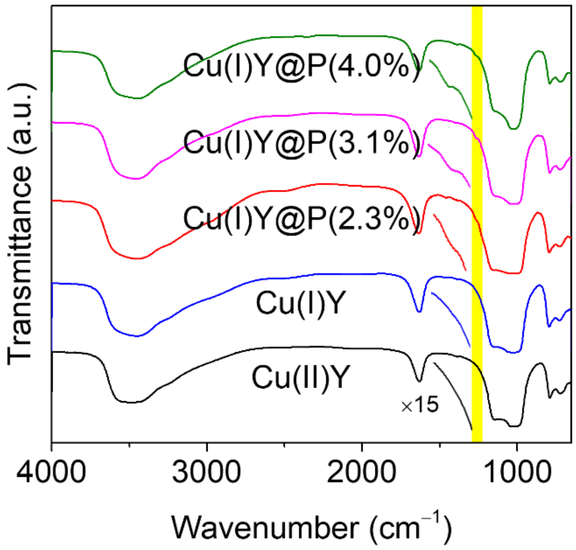

Supplementary Figure 6. FT-IR spectra of Cu(II)Y, Cu(I)Y, and Cu(I)Y@P.

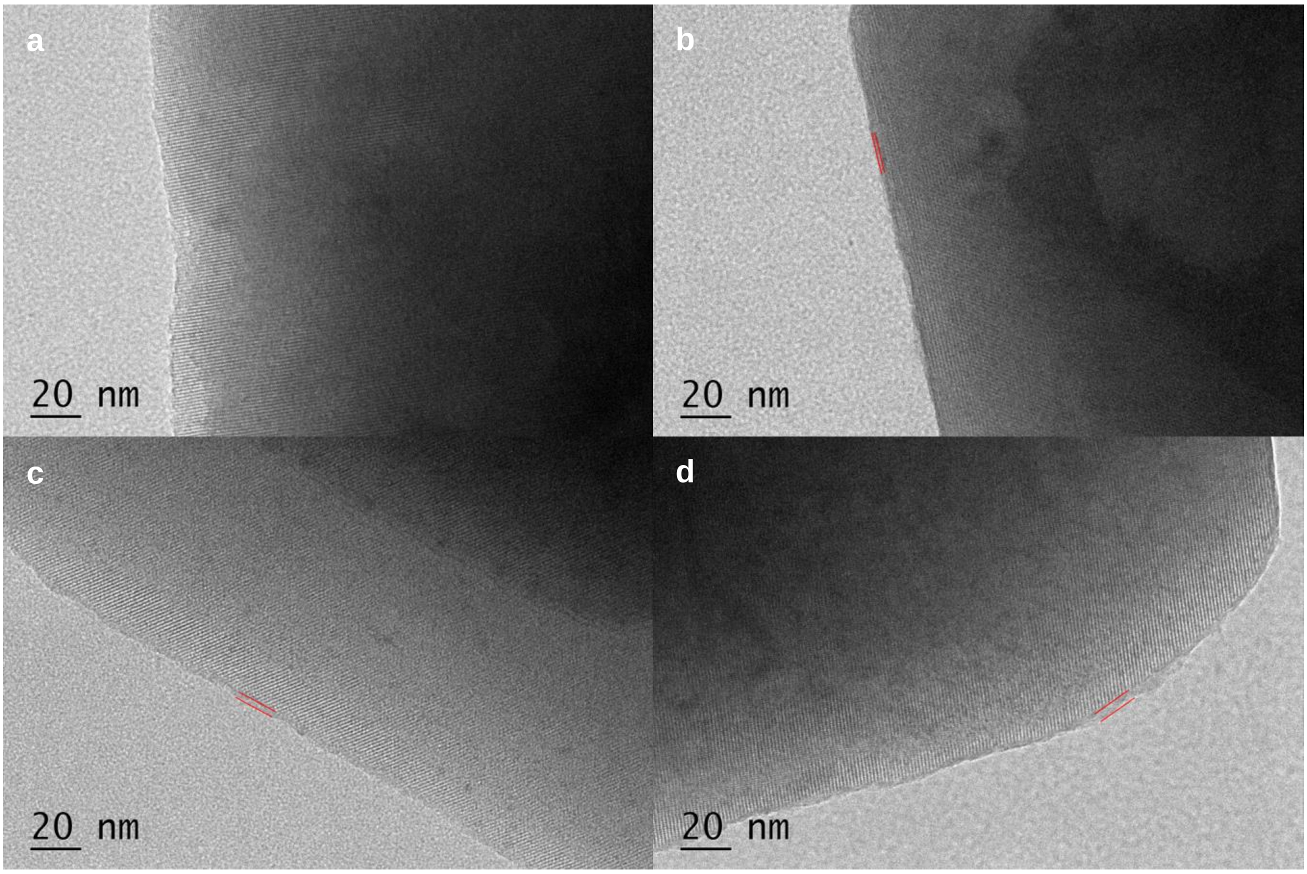

Supplementary Figure 7. TEM images of (a) Cu(I)Y, (b) Cu(I)Y@P(2.3%), (c) Cu(I)Y@P(3.1%), and (d) Cu(I)Y@P(4.0%).

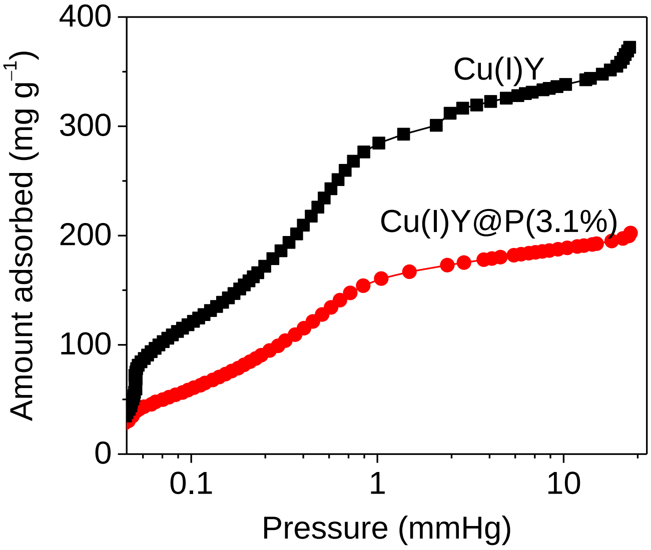

Supplementary Figure 8. Water sorption isotherms for Cu(I)Y and Cu(I)Y@P(3.1%) at 298 K.

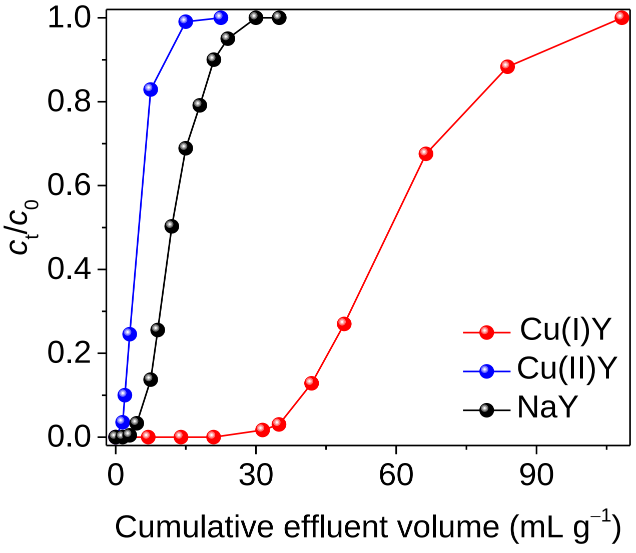

Supplementary Figure 9. Breakthrough curves of the model fuel containing 550 ppmw thiophene over NaY, Cu(II)Y, and Cu(I)Y.

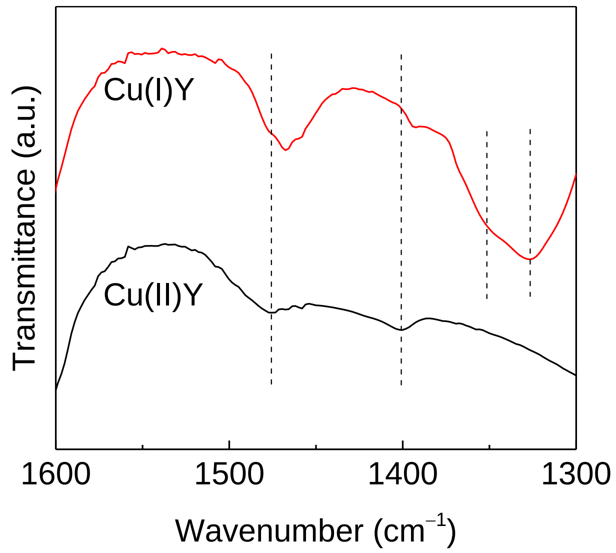

Supplementary Figure 10. IR spectra of thiophene adsorbed onto Cu(I)Y and Cu(II)Y.

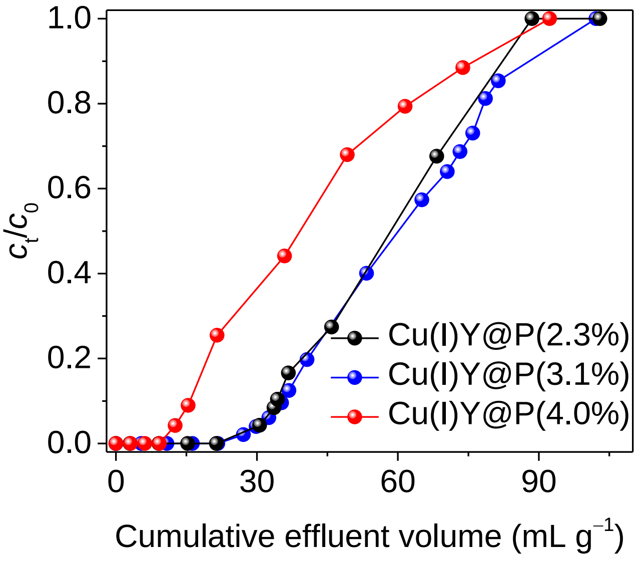

Supplementary Figure 11. Breakthrough curves of the model fuel containing 550 ppmw thiophene over Cu(I)Y@PDS.

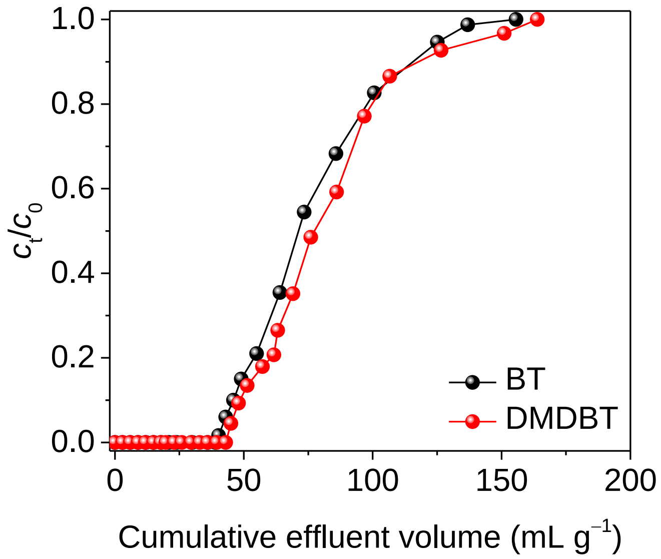

Supplementary Figure 12. Breakthrough curves of the model fuel containing 550 ppmw BT or DMDBT over Cu(I)Y@P(3.1%).

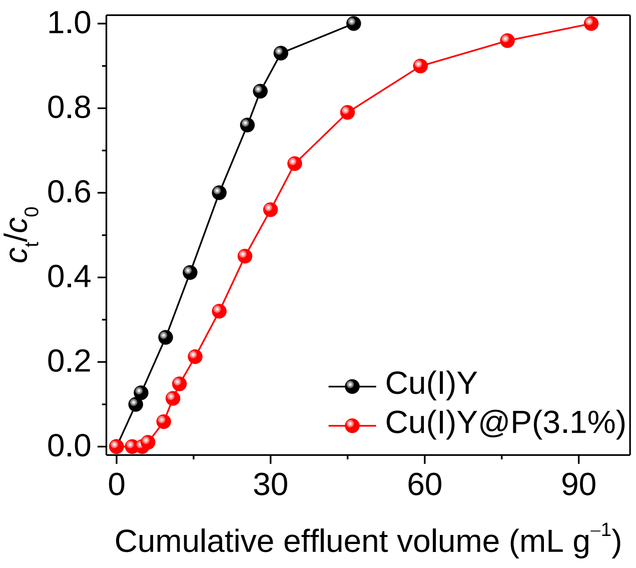

Supplementary Figure 13. Breakthrough curves of the model fuel containing 550 ppmw thiophene with the addition of 10 wt% of *tert*-butyl benzene over Cu(I)Y and Cu(I)Y@P(3.1%).

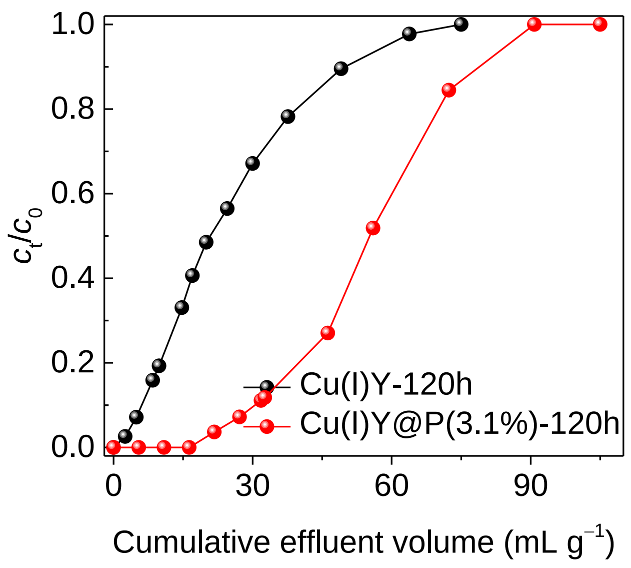

Supplementary Figure 14. Breakthrough curves of the model fuel containing 550 ppmw thiophene over Cu(I)Y and Cu(I)Y@P(3.1%) after exposure to humid atmosphere for 120 h.

Supplementary Tables

Supplementary Table 1. Physicochemical properties of different samples.

Samples	S_{BET} (m^2 g^{-1})	V_p (cm^3 g^{-1})	Elemental composition (wt %)	
			C	H
Cu(II)Y	793	0.34	-	-
Cu(I)Y	745	0.31	-	-
Cu(I)Y@P(2.3%)	744	0.31	0.73	2.44
Cu(I)Y@P(3.1%)	728	0.30	0.99	3.31
Cu(I)Y@P(4.0%)	410	0.23	1.25	4.22

Supplementary Table 2. Adsorption bond energies calculated from molecular orbital theory.[1,2]

Adsorbent	Adsorption bond energies (kcal mol^{-1})		
	Thiophene	BT	DMDBT
Cu(I) zeolite	21.4	22.9	23.6

Supplementary Table 3. Effects of moisture on the desulfurization capacity of the sorbents.

Adsorbent	Adsorption capacity for fuel without H_2O (μmol g^{-1})	Adsorption capacity for fuel with H_2O (μmol g^{-1})	Concentration of H_2O additive in the fuel	Ref.
Cu(I)Y@P(3.1%)	537	535	300 ppmw	This work
Cu(I)Y	548	244	300 ppmw	This work
Cu(I)Y(VPIE)	168[a]	26.5[a]	300 ppmw	[3]
AC-WPH	13.0	12.2	56 ppmw	[4]
Zn/Ni/Cu-BTC	30.0	14.5	500 ppmw	[5]
Zn55/Cu–BTC	24.8	22.1	water-saturated[b]	[6]

[a] Breakthrough capacity of sulfur. [b] Model fuel was mixed and thoroughly agitated with distilled water and then the water-saturated simulated oil can be obtained after getting rid of the water phase.

Supplementary Notes

Sample preparation

Cu(II)Y zeolite was prepared by ion exchange of NaY (Si/Al ratio of 4.86) with 0.5 mol L^{-1} $Cu(NO_3)_2$ aqueous solution at 90 °C for 48 h. The amount of Cu(II) in the ion-exchange solution was equivalent to 5-fold cation-exchange capacity. After ion exchange, the zeolite suspension was filtered and the solid was washed thoroughly, followed by dried at 100 °C overnight. The amount of Cu in zeolite was detected by inductively coupled plasma (ICP), and the results show that 79% of Cu was ion-exchanged, corresponding to a Cu content of 1.18 mmol g^{-1} and a Cu(I)/Al ratio of 0.24. The conversion of Cu(II)Y to Cu(I)Y was conducted by VIR. About 0.1 g Cu(II)Y was put in open vial and kept inside an autoclave containing about 1 mL methanol (CH_3OH) with no direct contact between the solid and the solution. The autoclave was then heated at 220 °C for 6 h. After the autoclave rapidly cooled to the room temperature, the vial with powder was taken out quickly, vacuumized by a Schlenk line to remove residual CH_3OH, and kept in the inert atmosphere. The obtained sample was denoted as Cu(I)Y. Cu(I) content in Cu(I)Y is influenced by the reduction time and temperature. By adjusting the reduction time and/or the reduction temperature, Cu(I) content can be increased to nearly 100%. Considering the desulfurization performance and energy cost, Cu(I)Y prepared under the current reduction conditions was selected for further investigation.

The PDS coating of Cu(I)Y was carried out by chemical vapor deposition (CVD) technique. Then Cu(I)Y was used for PDS coating, and thus the obtained Cu(I)Y@P materials possess the same ion-exchange ratio as Cu(I)Y. Alkyl-based or fluorinated compounds are commonly used to fabricate hydrophobic surfaces because of their ultralow surface energies. Some typical alkyl-based compounds include polystyrene, polyvinylpyrrolidone, and PDS and fluorinated compounds include

polyvinylidene difluoride and pentafluorobenzylamine. For the introduction of most hydrophobic compounds, pre-functionalization of the supports is required and then hydrophobic domains can be grafted. In addition, some hydrophobic domains are able to enter the pores of supports, leading to the decrease of porosity. It is worth noting that PDS can be coated on the support surface by vapor deposition with no special requirement on surface functionality; moreover, location of PDS on the outer surface is beneficial to the preservation of porosity because the large molecular weight of PDS would not enter the pores of zeolite. Therefore, we choose PDS over other types of hydrophobic materials to modify Cu(I)Y. Before coating PDS, Cu(I)Y was pretreated in flowing Ar at 400 °C for 30 min to remove adsorbed moisture. In the CVD process, a certain amount of Cu(I)Y (spreading to layer as thin as possible) was flat on glass dish, which was placed in a sealed glass container with some fresh PDS stamp. The glass container was maintained at 215 °C for a serious of different times varied from 15~45 min in a digital-temperature-controlled oven and then cooled to room temperature naturally to give Cu(I)Y@P(*n*), where *n* corresponding to the weight percentage of PDS calculated by elemental analysis.

General Characterization

XRD patterns of the materials were gained in the 2 θ range from 2° to 60° on a Bruker D8 Advance diffractometer with Cu Kα radiation at 40 kV and 40 mA. The N_2 adsorption isotherm was measured at –196 °C using an ASAP 2020 instrument. Ahead of analysis, the samples were degassed at 200 °C for 4 h under vacuum. The BET surface area was computed at relative pressure ranging from 0.04 to 0.25. The total pore volume was calculated from the amount adsorbed at a relative pressure of about 0.99. Water adsorption was investigated by using a volumetric adsorption apparatus (3Flex,

Micromeritics). The water contact angle was measured by static drop method on the model DROPMETER A-100P video optical contact angle measuring instrument. After pressing, the sample is placed on the platform and a drop of water is added to calculate the contact angle. The accuracy of water contact angle measurement is 0.1 degree. SEM was executed on a HITACHIS-4800 to observe the morphologies of the materials. TEM was executed on a JEM-2010UHR electron microscope at 200 kV. FT-IR measurements were performed on a NicoletS-4 Nexus 470 spectrometer using the KBr pellet technique. The spectra were gathered with a 2 cm^{-1} resolution. Adsorption of CO on adsorbents was investigated by low-pressure adsorption experiments monitored by Diffuse Reflectance Infrared Fourier Transform Spectroscopy (DRIFTS). The study was performed with a Nicolet NEXUS™ FT-IR spectrometer equipped with a liquid-nitrogen cooled MCT-A detector and an Ever-Glow mid-IR source and using a PIKE DiffuseIR™ diffuse reflectance accessory with a high-temperature environmental chamber equipped with KBr windows. A home-built pressure control system employing a turbomolecular pump and an electronically actuated leaking-valve was connected to the chamber. It allowed evacuation of the measurement chamber to a base-pressure of 2×10^{-6} mbar and adjustment of constant probe molecule pressure up to 100 mbar. Temperature-programmed desorption (TPD) experiments were conducted on a BELSORP BEL-CAT-A apparatus. About 50 mg of Cu(I)Y sample was pretreated at 150 °C under Ar for 6 h. After cooling to room temperature in an Ar atmosphere, the gas was switched to thiophene vapor with Ar. After adsorbed thiophene was purged by an Ar flow at room temperature, the sample was heated to 450 °C and the thiophene liberated was monitored continuously by a mass spectrometer (MS). X-ray photoelectron spectroscopy (XPS) analysis was carried on a Physical Electronic PHI-550 spectrometer equipped with an Al Kα X-ray source (hv=1486.6 eV) at 10 kV and 35 mA.

Quantitative analysis of Cu^+ content in the modified material was calculated by a wet chemistry titration method as reported in literature.[7] UV-vis spectra were gained using the Cintra 20 (Australian) spectrometer in the reflection mode. The spectra of samples were obtained in the wavelength range of 300–850 nm at intervals of 1 nm with a $BaSO_4$ reflectance standard used as the baseline. For the evaluation of the long-term stability of Cu(I), the Cu(I)-containing samples were placed in a container containing a saturated solution of NaCl at room temperature with RH=75% to accelerate the conversion of Cu(I). The exposed samples were characterized at different intervals.

Adsorption recycle experiments

Recycle experiments were carried out in situ on the breakthrough apparatus. The saturated adsorbent by model fuel was treated at room temperature for 2 h in an Ar flow, followed by heating at 150 °C for 30 min. The regenerated adsorbent was then cooled to room temperature for the desulfurization of model fuel again.

Supplementary Discussion

The formation mechanism for Cu_2O is explored as follows. Cu^{2+} in zeolite exist as $[Cu^{2+}OH^-]^+$ or $[Cu–O–Cu]^{2+}$ complexes.[8,9] The reducing agent CH_3OH moves into the gas phase from the liquid phase at elevated temperatures and diffuses into the pores of the zeolite. The redox reactions between CH_3OH and Cu(II) take place according to the following equations.

$$2[Cu^{2+}OH^-]^+ + CH_3OH \rightarrow Cu_2O + 2H^+ + HCHO + H_2O \quad (1)$$

$$[Cu–O–Cu]^{2+} + CH_3OH \rightarrow Cu_2O + 2H^+ + HCHO \quad (2)$$

In addition to Cu_2O, HCHO and H_2O are yielded as the gaseous products, which was detected by

mass spectrometer as reported previously.[10] H^+ along with Cu^+ work as the charge compensation ions for neutralizing negative charges emanating from the zeolite-lattice. The reduction of Cu(II) to Cu_2O as a separate phase in the zeolite lattice has also been reported previously.[11,12]

There is no Cu(0) formation confirmed from various characterizations such as XRD, UV, and XPS. Disproportionation of 2Cu(I) into Cu(0) and Cu(II) occurs in the presence of some *N*-containing ligands and polar solvents.[13] Under atmospheric environment, Cu(I) is easily oxidized to Cu(II) due to the synergistic cooperation between H_2O and O_2.[14,15] Therefore, there should be no Cu(0) formation under the current conditions of this study.

Considering the relatively small pore size of zeolite, the larger molecular size of DMDBT may result in slow diffusion. Nevertheless, slow diffusion hasn't been observed. Diffusion is actually related to both pore size and surface characteristic. It is reported that, due to the hydrophobic nature of PDS, appropriate PDS layer can promote the accumulation and diffusion of hydrophobic substrates.[16] Likewise, the diffusion of thiophenic sulfur compounds in isooctane is strengthened by PDS coating, which can accelerate adsorption process. Moreover, the pore size of zeolite can be well preserved when the coating amount of PDS is low. As a result, no decrease in diffusion is observed for the adsorbates with a larger molecular size.

Supplementary References

1. Yang, R. T., Hernandez-Maldonado, A. J. & Yang, F. H. Desulfurization of Transportation Fuels with Zeolites under Ambient Conditions. *Science* **301**, 79-81 (2003).
2. Hernandez-Maldonado, A. J. & Yang, R. T. Desulfurization of Transportation Fuels by Adsorption. *Catal. Rev.-Sci. Eng.* **46**, 111-150 (2004).

3 Li, Y. W., Yang, F. H., Qi, G. S. & Yang, R. T. Effects of Oxygenates and Moisture on Adsorptive Desulfurization of Liquid Fuels with Cu(I)Y Zeolite. *Catal. Today* **116**, 512-518 (2006).

4. Xiao, J., Song, C. S., Ma, X. L. & Lit, Z. Effects of Aromatics, Diesel additives, Nitrogen Compounds, and Moisture on Adsorptive Desulfurization of Diesel Fuel over Activated Carbon. *Ind. Eng. Chem. Res.* **51**, 3436-3443 (2012).
5. Wang, T. T. *et al.* The Remarkable Adsorption Capacity of Zinc/Nickel/Copper-Based Metal-Organic Frameworks for Thiophenic Sulfurs. *RSC Adv.* **6**, 105827-105832 (2016).
6. Wang, T. T., Li, X. X., Dai, W., Fang, Y. Y. & Huang, H. Enhanced Adsorption of Dibenzothiophene with Zinc/Copper-Based Metal-Organic Frameworks. *J. Mater. Chem. A* **3**, 21044-21050 (2015).
7. Jiang, W.-J. *et al.* Fabrication of Supported Cuprous Sites at Low Temperatures: An Efficient, Controllable Strategy Using Vapor-Induced Reduction. *J. Am. Chem. Soc.* **135**, 8137-8140 (2013).

8 Fowkes, A. J., Ibberson, R. M. & Rosseinsky, M. J. Structural Characterization of the Redox Behavior in Copper-Exchanged Sodium Zeolite Y by High-Resolution Powder Neutron Diffraction. *Chem. Mater.* **14**, 590-602 (2002).

9 Huang, S. Y. *et al.* Insight into the Tunable CuY Catalyst for Diethyl Carbonate by

Oxycarbonylation: Preparation Methods and Precursors. *Ind. Eng. Chem. Res.* **53**, 5838-5845 (2014).

10 Qin, J.-X., Wang, Z.-M., Liu, X.-Q., Li, Y.-X. & Sun, L.-B. Low-Temperature Fabrication of Cu(I) Sites in Zeolites by Using a Vapor-Induced Reduction Strategy. *J. Mater. Chem. A* **3**, 12247-12251 (2015).

11 Yu, J. S. & Kevan, L. Effects of Reoxidation and Water Vapor on Selective Partial Oxidation of Propylene to Acrolein in Copper(II)-exchanged X and Y Zeolites. *J. Phys. Chem.* **95**, 6648-6653 (1991).

12 Herman, R. G., Lunsford, J. H., Beyer, H., Jacobs, P. A. & Uytterhoeven, J. B. Redox Behavior of Transition Metal Ions in Zeolites. I. Reversibility of the Hydrogen Reduction of Copper Y Zeolites. *J. Phys. Chem.* **79**, 2388-2394 (1975).

13 Percec, V. *et al.* Ultrafast Synthesis of Ultrahigh Molar Mass Polymers by Metal-Catalyzed Living Radical Polymerization of Acrylates, Methacrylates, and Vinyl Chloride Mediated by SET at 25 °C. *J. Am. Chem. Soc.* **128**, 14156-14165 (2006).

14 Palomino, G. T. *et al.* Oxidation States of Copper Ions in ZSM-5 Zeolites. A Multitechnique Investigation. *J. Phys. Chem. B* **104**, 4064-4073 (2000).

15 Li, B., Ma, J.-G. & Cheng, P. Silica-Protection-Assisted Encapsulation of Cu_2O Nanocubes into a Metal-Organic Framework (ZIF-8) to Provide a Composite Catalyst. *Angew. Chem. Int. Ed.* **57**, 6834-6837 (2018).

16 Huang, G., Yang, Q. H., Xu, Q., Yu, S. H. & Jiang, H. L. Polydimethylsiloxane Coating for a Palladium/MOF Composite: Highly Improved Catalytic Performance by Surface Hydrophobization. *Angew. Chem. Int. Ed.* **55**, 7379-7383 (2016).
